# Pregnant racialised migrants and the ubiquitous border: The hostile environment as a technology of stratified reproduction

**DOI:** 10.1177/02610183231223951

**Published:** 2024-03-18

**Authors:** GWYNETH LONERGAN

**Affiliations:** 5995 Northumbria University, UK

**Keywords:** borders, hostile environment, migration, stratified reproduction

## Abstract

This article explores the impact of the ‘hostile environment’ on racialised migrant women's experiences of pregnancy and childbirth in England, arguing that the ‘hostile environment’ functions as a technology of ‘stratified reproduction.’ First coined by Shellee Colen, the concept of stratified reproduction describes the dynamic by which some individuals and groups may be supported in their reproductive activities, while others are disempowered and discouraged. This paper locates the stratified reproduction produced by the ‘hostile environment’ as intertwined with wider gendered and racialised discourses around British citizenship which have been ‘designed to fail’ racialised residents of the UK. Drawing on interviews with racialised migrant mothers in the north of England, this paper analyses how the proliferation and intensification of immigration controls interacts with gender, race, class, and other social regimes to differentially allocate the resources necessary for a safe and healthy pregnancy and childbirth, and how this is experienced materially by pregnant migrants.

## Introduction

‘Stratified reproduction’ was first conceptualized and developed by Shellee Colen (1995) to analyse how the state and wider social institutions differentially value and support the reproduction of certain individuals and groups. This paper explores the ‘hostile environment’ as a technology of stratified reproduction, specifically as it relates to the experience of racialised pregnant migrants and new mothers. It locates this reproductive stratification as intertwined with a wider set of policies, processes, and discourses around British citizenship which have been, as Tyler (2010) argues, ‘designed to fail’ racialised residents of the UK (see also Bassel and Khan, 2021; Erel et al., 2018; Solomos, 2003). The reproductive stratification produced by the hostile environment both reflects this racialised biopolitics of citizenship (Tyler, 2010); and illuminates the complexities and contradictions of its lived experience.

There has recently been a welcome increase in the attention paid by both the media (c.f. Kelly, 2022), and in scholarship (Feldman, 2021; Nellums et al., 2021) to the material consequences of charging un(der) documented pregnant migrants for maternity care. This paper explores the impact of hostile environment policies restricting migrants’ access to other welfare state services, and what this means for pregnant migrants and new mothers’ experiences of reproduction and reproductive tasks. The hostile environment is characterized by the proliferation, expansion, and intensification of bordering policies, that is, policies designed to construct and enforce the national border, e.g., visa checks (see, Reynolds et al., introduction to this special issue). The hostile environment subjects pregnant migrants and new mothers to multiple, overlapping, reinforcing immigration policies that interact with each other, and with gendered, racialised, and classed discourses of citizenship, and other social regimes, to produce multi-faceted patterns of reproductive stratification.

As outlined in the editorial introduction of this issue, the ‘hostile environment’ is associated with the changes to immigration policy enacted by the Coalition government, designed to make life difficult for (racialised) undocumented migrants. These new policies significantly expanded internal border checks in the UK; however, they should be read as continuous with, and building upon, legislation and policies of past governments, notably New Labour. Internal visa checks have been expanding and multiplying in the UK since the late 1990s. Indeed, legislation permitting visa checks in hospitals was first passed in 1982, although it was not implemented after a prolonged anti-racist campaign (Medien, 2021). The Immigration, Asylum, and Nationality Act 2006, for example, requires employers to undertake checks of their employees’ right to work in the UK, or face a penalty if they are found to be employing an undocumented worker. Moreover, it can be argued that asylum-seekers, especially, have long been subject to a ‘hostile environment’. Since 2002, for example, asylum-seekers have been banned from paid work except under narrow circumstances (Gower, 2021), and instead offered financial support through the National Asylum Support System (NASS), at less than the amount provided to unemployed UK citizens (Gov.uk, n.d.-a). NASS also provides asylum housing on a no-choice basis, on the condition that residents can be relocated at any time, a policy known as ‘dispersal.’ In recognition of these continuities, my exploration of the ‘hostile environment’ as a tool of stratified reproduction extends beyond the immediate policy changes enacted by the Coalition government.

The migrants interviewed for the project on which this paper is based were all cisgender women; and government policy, as well as gendered discourses around citizenship and reproduction, tend to construct pregnancy and childbirth as the exclusive experience of cisgender women. This paper, therefore, focuses on the experience of pregnant women, and this is reflected in the language used. Nonetheless, I do not wish to erase the experiences of pregnant trans men and non-binary people; indeed, research is urgently required on the interaction between transphobia and immigration policy in producing reproductive stratification, and its material consequences for pregnant migrants.

The paper begins by discussing the concept of stratified reproduction, and explores how immigration controls, underpinned by racialised and gendered discourses around citizenship, can serve to stratify support for reproduction and reproductive choice. Currently, this is being shaped by the multiplication and expansion of immigration policies and internal visa checks. I then discuss the methodologies used for data collection, before moving on to the empirical section of the paper. The expansive, proliferating nature of the hostile environment means that migrants are subjected to multiple, overlapping, reinforcing policies that stratify reproduction, and that interact with each other, and with racialised discourses around citizenship, in complex ways. Policies limiting certain ‘categories’ of migrant's entitlements to welfare state support interferes with their access to maternity care, and renders them vulnerable to living in housing that is unsuitable for babies and young children (Feldman, 2013). For people seeking asylum, this is aggravated by the policy of dispersal, which further interferes with access to adequate maternity care and disrupts support networks. The consequences of these policies are additionally exacerbated by Home Office bureaucracy and suspicion of migrants, but can be mitigated by the availability of local specialist midwifery support. As the hostile environment expands to encompass more and more areas of everyday life, its interference in, and stratifying effect upon, migrants’ reproduction similarly multiplies and intensifies.

## Stratified reproduction

The term ‘stratified reproduction’ was first coined to provide a framework for analysing how reproduction and reproductive tasks are ‘differentially experienced, valued, and rewarded according to inequalities of access to material and social resources in particular historical and cultural contexts’ (Colen, 1995: 78). Certain individuals’ reproduction is supported and nurtured by the state and wider social structures and institutions, while others’ is devalued and undermined (see also Bonizzoni, 2011; Ginsburg and Rapp, 1995b; Wu et al., 2019). Ginsburg and Rapp (1995b: 3), drawing on Colen (1995), understand stratified reproduction as additionally encompassing questions around ‘who is normatively entitled to refuse childbearing, to be a parent, to be a caretaker, to have other caretakers for their children, to give nurture or to give culture (or both)?’ Thus, stratified reproduction not only analyses whose reproductive practices are valued (or not), but whose reproductive *choices* are supported (or not) (see also Humphris, 2017; Moniz et al., 2022; Solazzo, 2019). Importantly, an individual may find their reproduction devalued and disempowered even as they are encouraged or pressured to carry out reproductive tasks for others; as Ginsburg and Rapp (1995b) point out, low-income African American women in the US may be stigmatised as ‘bad mothers’ who ‘burden’ the welfare system, even as, historically and at present, they are situated within a racially- and gender-stratified job market as carers for white women's children.

Scholars of stratified reproduction take an intersectional approach, arguing that support for reproduction and reproductive choices is stratified according to multiple, interacting, social structures and institutions, including capitalism, racism, gender, as well as state policies, operating at multiple scales, and within different national and local contexts (Beynon-Jones, 2013; Colen, 1995; Ginsburg and Rapp, 1995a, 1995b; McCormack, 2005; Smietana et al., 2018). In her work Colen (1995) explores how colonial legacies and neoliberal economic policies, enforced by transnational organisations like the World Bank, limit the opportunities for Caribbean women to earn enough money to raise their children in their countries of origin. They therefore migrate to the United States, where gendered and racialised discourses around reproduction, in combination with immigration policies, produce childcare as one of the few employment options that permit the women to earn a living, regularise their immigration status, and support their own children, whether in the US or overseas. Scholars have subsequently built upon Colen's work, analysing, for example: the interaction of ableism, race, and class in exploring why U.S. disabled women are more likely to be sterilised than their able-bodied peers (Wu et al., 2019); the ‘indirect’ impact of race/ethnicity on access to fertility support (Greil et al., 2011); and the complex, interacting, uneven, economic and social power relations underpinning LGBTQ persons’ access to, and use of, reproductive technologies, including surrogacy (Mamo and Alston-Stepnitz, 2015). Research around stratified reproduction also considers how intersecting discourses that differently value the reproduction of particular individuals and groups may be internalised by medical professionals and other gatekeepers, and the potential consequences, in different countries and contexts (Beynon-Jones, 2013; Goldade, 2011). Beynon-Jones (2013), for example, discusses the way in which health professionals providing abortions in Scotland construct patients’ requests as ‘rational’ or ‘problematic’ depending on a range of factors, including the patients’ age, class, and number of children, and how these locate the patients within discourses around ‘good’ motherhood. Moreover, stratified reproduction considers not only the way in which intersecting social structures and institutions interact in stratifying support for reproduction, and reproductive choices, but provides a *granular* analysis, exploring how these ‘macro-structural conditions’ impact, and are mitigated by, ‘microinteractions’, and the particular situation and circumstances of individuals (Mamo and Alston-Stepnitz, 2015: 521–522). Solazzo (2019) for example, discussing how race and class interact with legislation governing access to abortion in the United States, notes the additional impact of location, stating that ‘travelling even 25 miles to have the procedure is associated with later abortions, particularly among minority women.’ (539).

## Racialised citizenship, the expanding border, and stratified reproduction

Discourses around citizenship construct different individuals and groups as more or less capable of reproducing ‘good citizens’, and this is reflected in state policies that encourage or discourage reproduction along these lines (Erel, 2011; Gedalof, 2007; Ross, 2006). In the UK, for example, the policy of only paying child benefit for two children to families on universal credit (Department for Work and Pensions, 2017) can be read as discouraging the reproduction of low-income families. Because women are constructed as responsible for biological and social reproduction, policies and institutions dis/empowering and stratifying reproduction often disproportionately impact women (Erel, 2011; Gedalof, 2007; Lonergan, 2018; Luibhéid, 2013; Tyler, 2010). Furthermore, in the UK, discourses around citizenship and reproduction are heavily racialised (Bassel and Khan, 2021; Erel et al., 2018; Solomos, 2003; Yuval-Davis et al., 2019); indeed, Tyler (2010: 61–62) contends that UK citizenship can be understood as a biopolitics - ‘a field of biopolitical techniques and practices (legal, social, moral) through which populations are controlled and fashioned’ - that is ‘designed to fail’ along racialised lines. These techniques and practices demarcate and exclude racialised populations in the UK, producing them as precarious and/or ‘failed’ citizens (Tyler, 2010). Tyler's (2010) argument revolves around the 1981 British Nationality Act, which abolished Commonwealth migrants’ right to British citizenship, even as immigration policy at that time (and at present) provided paths to settlement for white people of British ancestry living abroad. This discursively and legally disassociated British citizenship from the wider empire, strengthening an already existing association between Britishness and whiteness, and producing racialised Commonwealth migrants as an ‘foreign’ population (Tyler, 2010; see also El-Enany, 2020; Medien, 2021). This racialised biopolitics of citizenship serves to produce reproductive stratification, as ‘successful citizens’ are supported in their reproductive decision-making and deemed fit to reproduce the nation; while the reproductive practises of ‘failed citizens’ are constructed as threatening the national future (Erel, 2018; Erel et al., 2018; Gedalof, 2007; Lonergan, 2018; Yuval-Davis et al., 2005).

Immigration policies both reflect, and reinforce, discourses around citizenship and belonging, with significant consequences for reproductive stratification (Colen, 1995). The racialised biopolitics of British citizenship means that racialised migrant women have fewer legal routes to entry and settlement, and that their reproductive practices may be especially devalued by the state and wider society (Bassel and Khan, 2021; Erel et al., 2018; Humphris, 2017; Lonergan, 2018; Yuval-Davis et al., 2005). As Tyler (2010) argues, the abolition of *jus soli* citizenship (whereby anyone born on a country's territory automatically becomes a citizen of that country) by the 1981 Nationality Act means that maternity wards are now sites where an alien population is introduced to the UK. This has contributed to the construction of (racialised) pregnant migrants as a target of hostile environment policies (Lonergan, 2023). Immigration policies additionally determine on what terms migrant families, or families with migrant members, can live together in a country; they also often differentially distribute access to welfare state resources necessary for reproductive tasks (Bonizzoni, 2011; Gedalof, 2007; Humphris, 2017; Lonergan, 2018).

The impact of immigration policies on reproduction and reproductive stratification has shifted over the last two decades as these policies have become increasingly strict and complex, and as internal border controls and visa checks have proliferated. While the ‘hostile environment’ is generally associated with the policies of the Coalition government, especially the 2014 Immigration Act, internal visa checks have been expanding in the UK since the Blair government, with asylum-seekers subject to particular policing and scrutiny (Bloch and Schuster, 2005), and members of the public co-opted into acting as border guards (Yuval-Davis et al., 2019). Yuval-Davis et al. (2019) argue that the experiences and impacts of this internal expansion of the border are situated and intersectional, as individuals are affected differently depending on their location within intersecting gendered, racialised, and classed discourses of citizenship and belonging, and according to the site at which visa checks take place. As immigration policies and visa checks proliferate, their influence on migrants’ experiences of reproduction will similarly intensify and expand; moreover, the situated and intersectional impact of these policies will further refine and entrench existing patterns of stratification along gendered, racialised, and class lines.

## Methodology

This article is based on a Wellcome-funded project exploring migrant women's experiences of pregnancy and maternity care in three areas of the north of England. Semi-structured interviews were conducted with forty-one migrant women who had been pregnant or given birth in one of the research areas. Most of the women interviewed had arrived in the UK in 2010 or after, and all had arrived in late adolescence or adulthood and so had not ‘grown up with the NHS’. Participants came from many different regions, including the Americas, Europe, the Middle East, South Asia and sub-Saharan Africa, and also had different immigration histories and visa statuses; among the women I interviewed were individuals who had come to the UK on work, spousal, and student visas; as dependents upon their spouse's visa; as EU migrants exercising their treaty rights (pre-Brexit); and as asylum-seekers and refugees. The diversity of the participant pool allowed me to explore how a range of factors might influence migrant women's experiences of pregnancy and maternity care. Thirty-two of the mothers interviewed were racialised migrants, and this paper focuses on their experiences. Where necessary, interviews were conducted with the support of an interpreter. Interviews also took place with sixteen NHS staff and five individuals who offer support to pregnant migrants as part of their third-sector job. In addition, I conducted two focus groups of three people with migrant interviewees; and held two end of project workshops with stakeholders, including migrants, NHS staff, and third sector employees and volunteers, one in-person, and one online. A selection of interview transcripts was initially coded by hand to identify key themes and develop two coding trees, one for interviews with migrants and migrant supporters, and one for interviews with NHS staff. Interview transcripts were then thematically coded using Nvivo 12, to assist with data management and the identification of broader patterns.

The Covid pandemic impacted both recruitment and the data collection process. Recruitment of migrants was conducted with the support of local charities working with migrants, who circulated information about the project with their service users, as well as through social media and word of mouth. Recruitment of NHS staff was conducted through NHS Trusts (the project was registered with Clinical Research Network Yorkshire) as well as local public health networks, the Royal College of Midwives, social media, and word of mouth. Recruitment of third sector interviewees was accomplished through existing networks. All interviews were conducted over the phone or on MS Teams due to the pandemic.

To ensure informed consent, when contact was made with prospective participants, a phone call was arranged, with the support of an interpreter where necessary, where the project was explained and the prospective participants given the opportunity to ask any questions. If the participant was interested, an interview date would be set, but with the clear caveat that the participant was able to withdraw prior to the interview, and for up to three months after the interview. Verbal consent was taken prior to the start of the interview and written consent at the end of the interview, when the participant could make an informed decision about whether they were happy with the process (see Lewis, 2015). Because of the pandemic, written consent was obtained either by posting or emailing the letter to the participant, who then returned it (if posted, a stamped envelope addressed to myself was included). Where appropriate, at the end of the interview, migrant participants were signposted to support services in their area. Because it can be more challenging to detect signs of distress when interviews take place over the phone or online, I made sure to make frequent enquiries regarding any interviewee's wellbeing and offered the opportunity for breaks or to end the interview if necessary. All participants have been anonymized and are referred to by a chosen pseudonym in this paper.

## The hostile environment and reproductive stratification beyond maternity charging

As discussed above, the proliferation of immigration controls and internal visa checks over the last two decades means that, depending on visa status, different ‘categories’ of migrants may be subject to multiple, overlapping, reinforcing policies with significant repercussions for their ability to access resources necessary for reproduction and reproductive tasks. The resulting reproductive stratification is further refined by the interaction of these policies with racialised, gendered, and classed discourses around citizenship and belonging, as well as the personal circumstances of individual migrants. Expanding internal visa checks have tended to target racialised persons, and their use of government services and other resources necessary for reproduction (Medien, 2021; Yuval-Davis et al., 2019).

The hostile environment serves to differentially restrict migrants’ ability to earn a living and/or receive benefits, depending on their immigration status. At the time of writing, the National Asylum Support System (NASS) provides persons with open asylum claims £40.85 per week. Pregnant women are entitled to an additional £3/week; babies under one years old, an additional £5/week; and children 1–3 years old an additional £3/week. In addition, pregnant women are eligible for a one-off grant of £300 if their baby is due in 8 weeks or less, or is under 6 weeks old. ‘Refused’ asylum-seekers can be made destitute; if they are destitute when they become pregnant, they can then apply for support, but only after 34 of weeks of pregnancy (Gov.uk, n.d.-b). Migrants on work, family, and student visas are subject to ‘No Recourse to Public Funds’ and therefore cannot collect benefits, but can (and indeed may be expected to) undertake paid work (UK Visas and Immigration, 2014). Undocumented migrants are legally forbidden from working, and may therefore be rendered destitute, or be subject to labour exploitation if they undertake paid work in the irregular economy (Bloch, 2013; De Genova, 2010). Marie, who works in the voluntary sector, argues that the limited support available to pregnant asylum-seekers reflects the lack of value placed by the state on these women's reproductive activities:

You know, they're entitled to Maternity Grants, they're entitled to £3 a week during pregnancy. I think, you know, we're looking at that group. The message loud and clear is like, a minimum amount of support, let's make it … you don't matter as much as other women … as much as other mothers. You don't matter, in the same way.

This devaluing of pregnant asylum-seekers can be read as an example of the racialised biopolitics of UK citizenship (Tyler, 2010) demarcating certain racialised populations, in this case migrant mothers, as ‘failed citizens’. However, as will be explored below, the lived experience of the resultant reproductive stratification is complex, as it is mitigated and mediated by a range of factors, including migrant women's own social and cultural resources, and the availability of local support networks.

### Maternity care

Hostile environment policies serve to differentially distribute access to the resources required for a safe and healthy pregnancy, and to raise children. Poverty caused by these policies can prevent some pregnant migrants from accessing antenatal and maternity care, irrespective of their legal entitlements (Phillimore, 2016). Helen, a specialist GP in Kirklees said of one of her asylum-seeking patients:

we couldn't get her to appointments, you know, she didn't have money, she couldn't access any money. It often comes down to the care is there on the NHS but getting people to it is not thought about.

Furthermore, Helen suggests that Home Office procedures and bureaucratic hurdles exacerbate the challenges caused by the low levels of support available on NASS:

there's an inflexibility in the Home Office funding, so [asylum-seekers] get their set amount of money and they get their accommodation but for example if they need to travel to a hospital that's further away and they can only get there by taxi, there isn't a fund you can access for that. They have to find their way somehow and although you can claim back hospital transport costs, it's complicated, and they want you to go on the bus and claim the cheapest …

For Michelle, one of my respondents who is seeking asylum, these financial barriers were further aggravated by Covid. Michelle had a baby during the Covid lockdown; the baby was very ill and had to spend some time in neonatal intensive care unit (NICU). Michelle was very worried about catching COVID on the bus, and so decided to take taxis instead to visit her baby in hospital, but these were prohibitively expensive, and she could not afford to visit her baby every day:

I have to take taxi every day, everything like that, but sometimes when there is no money anymore to take taxis, I have to leave it maybe two or three days before I go there, but I call, I call the people every day.

The stratified access to maternity care produced by policies impoverishing migrants is further refined and entrenched by NHS restructuring, and the personal circumstances of pregnant migrants, especially their place of residence. In recent years, specialist services, including maternity services, have tended to be concentrated in one hospital in a particular trust or area; for many pregnant women, this means they cannot access maternity services, especially obstetrics, in their local hospital. Alison, an obstetrician in West Yorkshire, said she felt that this specialisation, overall, allowed the maternity hospital to provide better services, but also discussed the way in which it can disadvantage low-income patients:

Interviewer: When you say that you think it might mean that people aren't able to access care, in what way?

Alison: Quite simply they might not be able to get to the hospital. You know, if you've got a baby that's not moving, and you've got to trek out of hours all the way to [hospital], and you live in [city at the other end of the trust area] it's a long way, isn't it?

Farah, who works in the third sector supporting migrant women, echoed these concerns; the organisation for whom she works is located in a large town 6 miles from the local maternity hospital, and she mentioned the cost, and the travel time, as challenges facing her clients when they try to attend their antenatal appointments.

Further stratifications around access to maternity care may be caused by discrepancies in the availability of specialist midwifery teams supporting vulnerable migrants, and individual midwives’ training and understanding of the impact of the hostile environment. Some of my asylum-seeking participants discussed the additional support they got from their midwife in navigating the hostile environment and applying for the funds to which they were entitled. Lisa, an asylum-seeker from South America, mentioned that while she felt her ‘regular’ midwife looked after her health well, she appreciated the specialist midwife's understanding of the asylum system and support she was able to provide:

Yeah, [the specialist midwife] helped me to write a letter to the Migrant Help [the charity contracted to oversee NASS support] for asking for the maternity grant … She helped me to write down with the details. She gave me the maternity certificate before the midwife from the NHS.

However, not all areas have a specialist midwifery service for vulnerable migrants, and consequently reproduction is further stratified along geographic lines. As well, racialised discourses stigmatising migrants, especially pregnant migrants, as illicitly using the NHS and other welfare state services may be internalised by midwives and other NHS staff, producing further stratification. Marie, discussing her work training NHS staff about migrant health reported that some midwives were keen to better support pregnant migrants, while others were somewhat hostile:

[O]n the whole it's mostly great, they're there because they want to understand someone's rights out there. And they get very confused about immigration. And why wouldn't they? Because it's so complicated, the system … But I have heard what I'd call sort of Daily Mail-y comments about scarce services being taken by people deliberately just coming here to have children and whatever, yeah.

### Housing

Hostile environment policies impoverishing migrants also serve as technologies of stratified reproduction by contributing to the differential distribution of safe and adequate housing for pregnant migrants and new mothers (Feldman, 2021). Two of the non-EU migrant mothers I interviewed found themselves in precarious housing situations produced by the interaction of the No Recourse to Public Funds policy with their gendered class position and the breakdown of their respective relationships. Asmani was able to collect Statutory Maternity Allowance due to her paid employment, but once her maternity leave was finished, she was unable to go back to paid work because she had no one to watch her child and because she was also caring for her mother, who was ill. Additionally, her relationship had broken down shortly after she became pregnant, and she was not supported, either financially or in terms of co-parenting, by the father of her child. Asmani wanted to move with her child out of her parents’ flat, where she was temporarily living, as it was very overcrowded, but the restrictions on her access to benefits initially made that impossible:

so horrible because I don't have any recourse to public funds and the maternity allowance finished and I don't have any money coming in, so where should I go? Nowhere. I need to stay there …

Humaira was pregnant with her third child and in the UK on a spousal visa when she decided, with the support of her midwife, to leave her abusive husband. After leaving her husband, Humaira went with her two children to the local council office, and was initially informed her that neither she, nor her two children, were entitled to council housing:

… they said because I have a spouse visa, my card was written like no access to public funds. Yeah, then they said; “I think we can't do anything for you because on your card is mentioned like you can't access any public funds.”

This caused Humaira considerable distress. After speaking with her social worker, the council agreed to house Humaira and her children, but they did so in a single hotel room, without laundry facilities or a kitchen. Interestingly, Humaira likely *was* entitled to housing, both because she was a victim of domestic violence (UK Visas and Immigration, 2019), and because her older children are British; her story thus also points to the further stratification caused by mistaken or overzealous gatekeepers.

Migrants with current asylum claims do not face homelessness in pregnancy because they are entitled to NASS housing – although they can be made homeless if their claim is refused – but the accommodation provided is notoriously terrible and sometimes unfit for safe pregnancy or mothering (Grayson, 2018). As Marie said in her interview:

And that is pretty, pretty poor, inadequate, accommodation. And for pregnant women, I think the main issues are, is this … is the type of support they get, and when they get it, and how they get it. And for those in antenatal care, what we see is a lot of - and this has got much worse during Covid - a lot of women who are being housed in full-board accommodation. Pregnant women.

‘Full-board’ accommodation here refers to women being housed in a room in a house with meals provided, rather than being given their own flat. Underlining Marie's words, one of my respondents postponed our scheduled interview because the mice running across the floor of the NASS flat she shared with her new baby kept her awake all night.

Both Covid and Home Office bureaucracy and inflexibility can exacerbate the reproductive stratification produced by inadequate asylum housing. Elior, an asylum-seeker, discussed in her interview the difficulties she experienced when she had to spend the Covid lockdown pregnant, with two children, in a one bedroom flat, because the Home Office erroneously believed her to have only one child:

Yes and especially my house is too tiny. The kids there is no place to go … sometimes my son will say mum, mum, I want to play, so sometimes it was, I used to get more emotion and especially when I was inside, like the flat is small, with my two babies and myself, I was thinking more when I was in detention, there's, the small, it was very difficult.

The mental toll of overcrowding was exacerbated for Elior by her memories of immigration detention – another example of one aspect of the hostile environment reinforcing the stratifying effects of another aspect. Although she managed to resolve the situation eventually, it required multiple phone calls to Migrant Help.

### Dispersal

Additionally, for pregnant migrants and new mothers in the asylum system, accommodation and financial support are tied to the policy of dispersal, which can serve as an instrument of stratified reproduction, disrupting continuity of antenatal care and removing a pregnant migrant from her support network (Feldman, 2013). The government has pledged not to disperse any migrants after 34 weeks of pregnancy; however Annie and Marie, who work in the voluntary sector, stated that the dispersal of pregnant women ‘is happening all the time’. Indeed, one of my respondents, Sana, was dispersed to a new city 6 days before she gave birth; in her interview, she discussed how it negatively affected her access to maternity care:

‘It was about six days left in delivery, I didn’t had any midwives, I didn’t knew any hospitals in [city], so it was just … it was a nightmare, I would say. But I was – I was struggling to get note from people that could guide me where to go.

Sana's experience is particularly striking given the emphasis on ‘continuity of care’ as best practice in NHS maternity care (National Maternity Review, 2016). Through the practice of dispersal, the UK government stratifies access to best practice in maternity care.

Dispersal, as well as the ban on working, can also produce reproductive stratification by making people seeking asylum especially vulnerable to isolation (Hynes, 2011; also see Stavroploulou in this issue). Similarly, precarious migrants, including refused asylum-seekers, may avoid socialising for fear they may ‘out’ themselves as un(der)documented (Bloch, 2014). This social isolation can interfere with pregnant migrants’ access to, and experiences of, maternity care, regardless of legal entitlements. Sana had to give birth without her husband's presence; having recently moved to their new city, they did not have any friends who could watch their other children. It was also reported by respondents that pregnant migrants who were asylum-seekers or otherwise had precarious immigration status were skipping antenatal appointments because they had no one to watch their children (see also Phillimore, 2016); as Farah put it:

If they have got another child in a school, somebody has to pick the children up. If the appointment is delayed, they are worrying nobody is going to take the child at home.

As with the challenges facing impoverished migrants in accessing maternity care, local services, and especially local specialist midwives, may be able to mitigate the harmful impact of poor housing and dispersal. Salma, a midwife who works with vulnerable families, described the specialist support she provides for an asylum-seeking pregnant woman:

So this morning, for example, I’m being [audio cuts out] to an Afghan refugee who's moved into a bridging hotel here in [city] … And so I’ll be booking her into our services. So I’ll be doing a full clinical booking to make sure that they know … because there's a potential for care to be fragmented, we might not have all the information that we need. Make sure we meet her where she is, really.

However, as not all areas have a specialist midwife services, and not all midwives understand the challenges facing precarious migrants. This can, as noted previously, produce further reproductive stratification according to geographic location, especially in interaction with racialised discourses stigmatizing pregnant migrants. Jill, who works with destitute migrants, for example, relayed the experience of one of her clients, an asylum-seeking mother living in NASS accommodation:

[The client] was complaining to the midwife, when they visited her at home, but she was living in a grotty B&B, and she was saying “I’m having to wash the bottles in the sink in the bathroom bit.” And the midwife just said, “Well, you’re from Africa.”’

### The lack of home office accountability

The stratifying effect of all of these policies upon reproduction is intensified by the suspicion and surveillance of migrants characteristic of the hostile environment, as well as the lack of accountability within the Home Office. As mentioned, Elior's situation was caused by a Home Office clerical error, and it took several phone calls to Migrant Help, and several months, to resolve. Asmani discovered, through her own research, that she might be eligible for special consideration given the change in her circumstances and asked a friend to write to the Home Office on her behalf. In her interview, Asmani discussed the Home Office's initial response, which exemplifies both the severity of NRPF and the surveillance to which migrants are subjected:

[The] Home Office sent me the letter, they said, your bank statement [which she was required to submit] shows that you are spending money on high street and I just thought, really? Did you give me any money before? Did I claim any benefits before? It was my money. I earned it. I got maternity allowance on my work

Jill, who works with destitute migrants, also discussed her experience attempting to secure financial support for her pregnant clients, and the impact bureaucracy and lack of accountability in the Home Office:

I think the biggest frustration is the Home Office not responding to things. So we’ll submit stuff, and you don’t hear back for weeks. So we’ll make an application for a maternity grant and spend our lives chasing it up.

Jill goes on to say that this lack of accountability can be especially stressful for destitute pregnant migrants waiting to hear if their application for support has been successful:

The uncertainty is a massive thing. If it's someone who's not on support, it's the waiting till the point where they can apply for support, it's really late on in the pregnancy, and then not knowing where they’re going to be dispersed to or what the accommodation's gonna be like.

The hostile environment therefore subjects pregnant migrants, depending on their visa status, to multiple overlapping policies which limit their access to resources necessary for safe and healthy reproduction. These policies interact with racialised, gendered, and classed discourses around citizenship and belonging, as well as the personal circumstances of individual migrants, to further produce reproductive stratification, which is then additionally reinforced and refined by the suspicion, bureaucracy, and lack of accountability characteristic of the hostile environment. As the hostile environment expands, so too does its influence on myriad aspects of reproduction and reproductive activities. Farah summarised the way in which the multiple consequences of the hostile environment, including financial and housing precarity, social isolation and anxiety and uncertainty, means some of her clients are simply unable to prioritise their pregnancy:

because they got lots of difficulty … for example the paperwork, they haven't got their status. So they have got all this worry to think about, so they kind of push themselves at the end of the queue, you know. This pregnancy is not important … … I don't mean they don't care about it, but they have got bigger deals, because- because no money, they have got no money. Money to feed the children, taking children into school. They haven't got shelter.

## Conclusion

Hostile environment policies serve as technologies of stratified reproduction, differentially distributing access to resources critical to reproduction. As immigration policies multiply and proliferate internally, their impact on the reproductive experiences of pregnant migrants and new mothers similarly expands. Pregnant migrants can be subject to multiple, overlapping policies that exert influence over myriad aspects of reproduction, including their ability to access medical care, housing, and a basic income. Moreover, these policies interact with racialised, gendered, and classed discourses around citizenship and reproduction, as well as the personal circumstances of individual migrants, to produce further reproductive stratification. As with the impact of internal borders more broadly, experiences of reproductive stratification created through immigration controls are situated and intersectional. Racialised migrants are more likely to experience reproductive stratification because of hostile environment policies, both because racialised immigration controls provide fewer routes to permanent, legal settlement for these migrants, and because gatekeepers, including midwives, may internalize racialised discourses depicting these migrants as illicitly using the NHS and other welfare state services.

The reproductive stratification produced by the hostile environment can be read as an aspect of the wider racialised biopolitics of UK citizenship analysed by Tyler (2010). Discourses and practices mark out racialised migrant women as an outsider population whose reproductive practices are threatening to the state, and must therefore be disciplined (Erel, 2018; Erel et al., 2018; Gedalof, 2007; Lonergan, 2018, 2023; Tyler, 2010; Yuval-Davis et al., 2005). Reproductive stratification thus emerges as deeply intertwined with citizenship, and with strategies to demarcate and control certain populations as ‘failed’ citizens. At the same time, however, the findings discussed in this paper point to the complexity and contradictions of the lived experience of the racialised biopolitics of citizenship. My research participants’ experiences of the impact of hostile environment policies on their reproductive autonomy were mediated by their own individual situations, as well as the availability of local sympathy and support, whether through midwives or third sector organisations.

This paper explores only a few aspects of the role of the hostile environment in producing reproductive stratification; the impact of family migration policies on racialised migrants’ experiences of reproduction, and the way in which these policies construct certain families’ reproduction as more valuable than others, for example, is not discussed; nor are the experiences of transgender and nonbinary pregnant migrants, and the interaction between transphobia and bordering processes in producing reproductive stratification. As Ross (2006) argues, policies around immigration and those around reproduction are both, ultimately, about who can be a citizen, and on what terms. The proliferation and multiplication of immigration policies under the hostile environment will inevitably increase state interference in, and stratification of, reproduction. Scholarship must remain sensitive to changing dynamics and consequences of this stratification, in order to understand which groups may become targeted as ‘failed citizens’, and how the marginalisations and exclusions that both produce, and result from, reproductive stratification, are imposed and experienced.
